# Open innovation for entrepreneurial opportunities: how can stakeholder involvement foster new products in science and technology-based start-ups?

**DOI:** 10.1016/j.heliyon.2022.e11897

**Published:** 2022-11-28

**Authors:** Patricia P. Iglesias-Sánchez, Alain Fayolle, Carmen Jambrino-Maldonado, Carlos de las Heras-Pedrosa

**Affiliations:** a Department of Economics and Business Organization, Universidad de Málaga, 29071 Malaga, Spain; b CREA University of Cagliari, Italy and Entrepreneurship Research Centre, EM Lyon Business School, Lyon, France; c Department of Audiovisual Communication and Advertising, Universidad de Málaga, 29071 Malaga, Spain

**Keywords:** Open innovation, Stakeholder, Start-ups, Opportunities, New product development, Innovativeness, Entrepreneurship

## Abstract

An open innovation approach can contribute to management opportunities. This article analyses how Science and Technology-Based Start-ups (STBSUs) achieve entrepreneurial opportunities involving stakeholders. The basis of the analysis comes from a qualitative and exploratory study of 24 STBSUs from two entrepreneurial ecosystems in France and Spain. The empirical data shows the active listening and generation of a co-creation environment where stakeholders emerge as key tools for innovativeness, competitiveness and survival in STBSUs. The findings suggest the recognition of uncertainty is a driving force for implementing open innovation. The findings contribute empirical insights into how STBSUs take advantage of stakeholder contribution in product development and suggest practical implications and future directions in the field of entrepreneurship and open innovation.

## 1 Introduction

Business success is undoubtedly linked with entrepreneurial opportunities (hereafter EO) (Alvarez and Barney, 2007; Sarasvathy, 2014). Previous empirical literature has widely focused on this process for science and technology start-ups (STBSUs) (Alvarez et al., 2016; Foss and Klein, 2017; Gruber et al., 2013; Hasan and Koning, 2019; Jones and Barnir, 2019; Park, 2005; Secundo et al., 2020), and managing their innovation and ensuring their survival is viewed as a continuous challenge (Companys and McMullen, 2007; Grama-Vigouroux et al., 2020; Michelino et al., 2017; Spender et al., 2017). Furthermore, in recent years, the open innovation (OI) approach, introduced by Chesbrough (2003), has gained traction among practitioners and researchers. Both agree that openness to external sources of innovation can contribute to increased competitiveness (Jones and Barnir, 2019), and there are several research works that emphasise the key role of stakeholders with regards to EO (Alberti and Pizzurno, 2017; Bogers et al., 2017; Chesbrough and Bogers, 2014; Eiteneyer et al., 2019; Grimaldi et al., 2013; Ollila and Yström, 2016; Urbinati et al., 2021) and the potential of collaborative innovation involving customers, suppliers, universities, or others in the process (Danarahmanto et al., 2020; Eftekhari and Bogers, 2015; Lassen and Laugen, 2017; Neyens et al., 2010; Urbinati et al., 2021).

The expectancy generated by OI in STBSUs has provided a substantial body of literature (Ardito et al., 2020; Marullo et al., 2018; Spender et al., 2017). In fact, many advances have been made in terms of understanding the process and motivation of its implementation (Bogers et al., 2017; Danarahmanto et al., 2020; Spender et al., 2017; Xie and Wang, 2020). There is extensive consensus regarding the close relationship between the start-up phenomenon and OI (Spender et al., 2017). Furthermore, it is evident that OI adoption is suitable for STBSUs to overcome some of the weaknesses related to their characterisation (Danarahmanto et al., 2020; Eftekhari and Bogers, 2015; Greco et al., 2016; Kirchberger et al., 2020; Marullo et al., 2018; Michelino et al., 2017; Sandmeier et al., 2010; West and Bogers, 2014). However, as Hasche et al. (2017); Kirchberger et al. (2020) and Xie and Wang (2020) state, further research is required since several issues remain unresolved. On the one hand, Michelino et al. (2017) emphasised the lack of empirical findings to understand the processes, and enablers for implementation, especially in the case of STBSUs. However the above mentioned issues have been widely discussed with respect to other types of companies (Ibidunni et al., 2020). On the other hand, integrating stakeholder involvement as a key strategy in the OI approach also requires a complete understanding of EO management to explain new product development (NPD) and business model definitions (de Oliveira and Cortimiglia, 2017; Xie and Wang, 2020). This study focuses on how OI, through stakeholder involvement, contributes to better EO management for STBSUs based in France and Spain. Therefore, the underlying research questions of our study are as follows: (1) How can STBSUs’ stakeholder involvement—as the core of the OI approach—aid EO emergence? (2) To what extent does OI strategy through stakeholder involvement foster NPD and contribute to business model definition?

Based on the above, STBSUs may be particularly prone to adopting this approach. These companies constitute a special case to study how EO is managed as a fundamental basis of innovation and competitive strategy, and to evaluate how much OI contributes. Furthermore, as a differentiating element, the analysis of the establishment of these strategies is made paying attention to stakeholder involvement as a way of implementing this approach.

The main contributions are concerned with the standardisation of the OI strategy in STBSUs in the daily challenge of engendering EO and the key role of stakeholder involvement in achieving expected outputs of innovation levels, competitiveness, and survival. Consequently, it allows the advancement of the theoretical framework with empirical evidence and with specific practices. Furthermore, this paper shows stakeholder involvement as an efficient and common mechanism in order to overcome the high degree of uncertainty associated with STBSUs. In fact, it is highlighted how their implementation fosters new product development, and consequently expected outputs of innovation levels, competitiveness, and survival are achieved.

The remainder of this paper is structured as follows. Section 2 covers a brief literature review, followed by Section 3, which describes the methodology of the study. Next, in Sections 4 and 5 we present the results of our analyses and discuss the findings, respectively. Section 6 concludes and provides the contributions and limitations of the research.

## 2 Theoretical framework

### 2.1 Entrepreneurial opportunities as a source of innovation in science and technology start-ups

Researchers and practitioners have continued to analyse how EOs are managed (Alvarez et al., 2016; Foss and Klein, 2017; Jones and Barnir, 2019). New ventures need to conceptualise their business models from the perspective of an opportunity (Alvarez and Barney, 2007; de Oliveira and Cortimiglia, 2017; Sarasvathy, 2014). Entrepreneurial opportunities are essential for their development (Gruber et al., 2013) and to ensure their survival (Companys and McMullen, 2007). Furthermore, EOs facilitate identifying a source of ideas for setting up a company or developing new products and services. Moreover, according to Shane et al. (2010), just one technology or innovation can result in several EOs, and this is precisely the challenge for entrepreneurs (Park, 2005).

However, Entrepreneurial opportunities are the starting point of innovation, but are fruitful only if they create value for customers (Danarahmanto et al., 2020; Keinz and Prügl, 2010; Ollila and Yström, 2016). Thus, it is necessary to allocate time and attention to search for such opportunities (Gruber et al., 2013; Hannigan et al., 2018) and consider the strategies, tools, or practices that make it easier to face this challenge (Chesbrough and Bogers, 2014; Hasan and Koning, 2019; Wouters, 2010; Xie and Wang, 2020).

Entrepreneurial opportunities are one of the keys to success for all enterprises, and in particular for companies whose core is innovation, such as STBSUs. Eftekhari and Bogers (2015) stressed that STBSUs are an important source of innovation in any economy and country. Michelino et al. (2017, p. 12) defined them as follows: “Start-ups are new enterprises in the first stage of their operations, working to solve a problem, where the solution is not obvious and the success is not guaranteed. This business is typically technology-oriented and has a high growth potential” (Michelino et al., 2017, p. 12). Consequently, it is clear that STBSUs depend on their ability to manage insights on which to base their business models, and thus they require continuous innovation (Danarahmanto et al., 2020; Park, 2005; Urbinati et al., 2021). However, many issues should be explored to better understand how these companies manage opportunities in order to achieve their level of innovation and be competitive and sustainable. These pending matters are pointed out by Hasche et al. (2017), Hannigan et al. (2018), Jones and Barnir (2019), Secundo et al. (2020) and, consequently, this gap should be filled by encouraging the development of research such as this.

### 2.2 The role of external collaboration on innovation

Innovation is a dynamic and complex process that depends on several factors and multiple players (Alberti and Pizzurno, 2017; Grama-Vigouroux et al., 2020). The focus on influential actors has attracted the attention of researchers and practitioners (Kirchberger et al., 2020).

Consequently, in recent years, this has resulted in prolific literature linking innovation and networks, and specifically, the role of stakeholders in this process (Danarahmanto et al., 2020; Lassen and Laugen, 2017). Competitive advantage is achieved as a result of this collaboration (Danarahmanto et al., 2020; Fang, 2008; Randhawa et al., 2021) between companies between countries (Ahn et al., 2017). Therefore, the key is the recognition and exploitation of opportunities that arise from the alignment of internal capabilities with external knowledge (Ardito et al., 2020; Chesbrough, 2003; Eftekhari and Bogers, 2015; Ibidunni et al., 2020; Park, 2005).

Nevertheless, the management of relationships with stakeholders is essential to reach a desired outcome (Eiteneyer et al., 2019; Lassen and Laugen, 2017; Sarasvathy, 2014; Vanhaverbeke and Roijakkers, 2015). A substantial body of literature highlights the effectiveness of networks on innovation (Alberti and Pizzurno, 2017; Bogers et al., 2017; Chesbrough and Bogers, 2014; Danarahmanto et al., 2020; Eiteneyer et al., 2019; Ferriani and MacMillan, 2017; Grimaldi et al., 2013; Ollila and Yström, 2016). Specifically, external collaboration provides knowledge, risk reduction, speed of development (Eftekhari and Bogers, 2015; Grama-Vigouroux et al., 2020; Sandmeier et al., 2010), and even supports firms' business models (de Oliveira and Cortimiglia, 2017; Mary George et al., 2016; Xu and Koivumäki, 2019) and sustainable performance (Danarahmanto et al., 2020; Fang, 2008; Ibidunni et al., 2020). There is a broad consensus that managing the firms’ boundaries is beneficial for competition (Ardito et al., 2020; Lassen and Laugen, 2017; Randhawa et al., 2021), and it should be a priority for the success of STBSUs (Keinz and Prügl, 2010).

According to Eftekhari and Bogers (2015) and Xie and Wang (2020), ecosystem collaboration, customer involvement, and OI are keys to successful implementation. The alignment of these principles facilitates the exploration and exploitation of opportunities that create innovation in businesses (Bogers et al., 2017; Chesbrough, 2003; Randhawa et al., 2021). By extension, STBSUs use networks to innovate in practice. Accordingly, it should be emphasised that the STBSUs are active in innovation ecosystems and involve stakeholders in order to overcome their weaknesses and survive (Eftekhari and Bogers, 2015).

Despite some contending references, this topic has often been investigated with ambiguous and contradictory results over time (Spender et al., 2017). Furthermore, in the previous literature, it is not clear what collaborations are more valuable: customers, suppliers, universities, providers, or partners, amongst others. This issue has garnered attention in recent years. For example, Lassen and Laugen (2017) concluded that customers and suppliers are the most involved, while competitors, universities, and other networks are involved to a lesser extent. Likewise, Spender et al. (2017) proposed that incubators, large corporations, other start-ups and entrepreneurs, and higher education systems are more relevant.

Accordingly, STBSUs encourage links with scientific organisations, especially with universities and research centres, due to their characterisation (Michelino et al., 2017). Other studies have highlighted customers as co-creators of value and defined them as a key ally for a firm's value proposition as compared to other stakeholders (Danarahmanto et al., 2020; Zalewska-Kurek et al., 2016). It is noteworthy that there seems to be a correlation between the kind of innovation—incremental or radical—and the different types of stakeholders (Ardito et al., 2020; Neyens et al., 2010).

Likewise, the number of ties has also been emphasised (Lassen and Laugen, 2017). However, there are more correlations between the duration of an alliance than the specific actors in STBSUs’ innovation performance (Laursen and Salter, 2006; Neyens et al., 2010). Even, the centrality of networks deserves particular focus (Ferriani and MacMillan, 2017).

The challenge is the management of the relationships with stakeholders; for example, Danarahmanto et al. (2020), Greco et al. (2016) and Randhawa et al. (2021) perceived customers as powerful stakeholders that create value; however, there is no consensus between the authors about costs concerning maintaining and creating relationships with customers (Ferriani and MacMillan, 2017; Subtil de Oliveira et al., 2018).

Consequently, these companies are often not sufficiently developed in such areas (Michelino et al., 2017). Following the aforementioned literature, the first research question of this study aims to fill the gaps due to the scarcity of studies that have focused on exploration of):

RQ1 How do STBSUs involve stakeholders in entrepreneurial opportunities?

### 2.3 The challenge of stakeholder involvement through open innovation approach in STBSUs

Open innovation continues to generate research interest, and there are many questions that remain unanswered (West and Bogers, 2017; Xie and Wang, 2020) since its introduction by Chesbrough (2003). The basis of the OI approach is “the use of purposive inflows and outflows of knowledge to accelerate internal innovation and expand the markets for external use of innovation, respectively” (Chesbrough and Crowther, 2006, p. 1). A wide range of parties is considered, so stakeholders are accounted for according to the idea of innovation funnel boundaries. In this case, innovation is understood as EOs that turn into new products or services, or even define or change the business model for STBSUs. It seems that these innovation dynamics allow STBSUs to gain competitive advantage and performance and maintain their alertness for new opportunities, innovation models, and so on (Alberti and Pizzurno, 2017; Spender et al., 2017; Xie and Wang, 2020). Open projects in which stakeholder involvement is implemented are strongly associated with OI (Eiteneyer et al., 2019; Spender et al., 2017; Urbinati et al., 2021; West and Bogers, 2017).

The theoretical framework of OI underscores that external collaboration is an effective formula to achieve the best innovation outcomes (Eiteneyer et al., 2019; Lassen and Laugen, 2017). On the other hand, extant literature agrees that stakeholder involvement is a common pattern to implement regarding OI (Danarahmanto et al., 2020; Marullo et al., 2018; Michelino et al., 2017; Ollila and Yström, 2016; Secundo et al., 2020; Spender et al., 2017; Xie and Wang, 2020). There is a common ground between OI and EOs through stakeholder involvement as a strategy of innovation management, which is the focus of this study. The literature in both fields provides evidence that collaborative innovation is a success factor for entrepreneurs (Grama-Vigouroux et al., 2020; Marullo et al., 2018) despite the scarcity of analysis exploring the influence these have on each other, it means opportunities emerge thanks to OI.

However, empirical results regarding how stakeholders contribute to new product or service development to engender a more competitive business model have not been fully addressed, especially in the case of STBSUs. Specifically, Lassen and Laugen (2017) warned about the direct relation between innovation and some contextual factors such as the technological complexity typical of targeted STBSUs, while Spender et al. (2017, p. 4) stressed in particular that ‘Start-up companies represent a powerful engine of OI processes’.

Several recent studies have shown that STBSUs follow OI strategic direction (Alberti and Pizzurno, 2017; Hasan and Koning, 2019; Marullo et al., 2018; Vanhaverbeke and Roijakkers, 2015). The main reasons for the increasing importance of OI for these firms are: overcoming resource constraints owing to their newness and size (Eftekhari and Bogers, 2015; Greco et al., 2016; Marullo et al., 2018; West and Bogers, 2014), and the uncertainty STBSUs face on a daily basis (Hasan and Koning, 2019; Secundo et al., 2020).

Several authors (Chesbrough, 2003; Eftekhari and Bogers, 2015; Sandmeier et al., 2010; Wouters, 2010; Xie and Wang, 2020) have highlighted the lack of information about the market and existing customers in the novel context of STBSUs' products and services. Consequently, STBSUs operate in an uncertain scenario that encourages them to be more open and collaborative with their stakeholders (Bogers et al., 2017; Kirchberger et al., 2020; Sarasvathy, 2014; Xu and Koivumäki, 2019). Accordingly, Michelino et al. (2017) stated that “start-ups may overcome their business limits and circumstances by forming relationships with external partners and organisations. Such relationships improve the quality of start-ups’ products and services and positively affect their business models” (Michelino et al., 2017, p. 113). To understand how OI strategies are implemented for EO management and the resulting outcomes, we proposed the following research question:

RQ2 To what extent does OI strategy through stakeholder involvement foster NPD and contribute to business model definition?

## 3 Material and methods

A qualitative study based on a multiple case study approach was developed to obtain a better understanding of the multiple interactions, strategies, and characterisation of STBSUs and the constant challenge of EO management. The appropriateness of a qualitative study with a multiple-case approach is supported by Eisenhardt and Graebner (2007) who highlight the capacity of the empirical evidence from one or more cases to create propositions and understand a phenomenon. Moreover, it allows the identification and better understanding of patterns, differences and, even, causal relationships, in this case through an exploratory analysis of the adoption of the OI approach with the support of stakeholders STBSUs. Studying several cases in detail allowed the research group of this study to gain knowledge of the relevant settings, strategies, and motivations (Sandmeier et al., 2010; Yin, 1994). In this case, the international comparison was valuable for the analysis because of the corroboration and triangulation of data (Yin, 2014).

### 3.1 Sample

We built on 24 cases comprising 12 STBSUs linked with the entrepreneurial ecosystem in France and 12 STBSUs linked with the university entrepreneurial ecosystem in Spain. The suggestion for applying case analyses is a range from four to ten, according to Eisenhardt (1989). However, the robustness of the analysis should be emphasized because the number of cases was intended to be as representative as possible of the total number of start-ups in each ecosystem, despite the qualitative method. Based on the total number of start-ups set-up and active, the number of STBSUs interviewed for the study represents 30% of the total in both ecosystems. STBSUs from two very similar university-based entrepreneurial ecosystems were selected. To form a unique research sample composed of sub-samples from these two European countries, in the first phase of the research, the directors of the incubators in the two entrepreneurial ecosystems were interviewed in order to fully understand the potential effects of contextual and institutional factors.

It should be emphasised that the study used a purposive sample. This means that the units of investigation that were relevant to the study were selected (Patton, 2001). Eisenhardt and Graebner (2007) defended the usefulness of theoretical sampling by its capacity to support the relationships and logic among constructs. It is preferred to random or stratified sampling in exploratory studies with relatively unknown phenomena.

The sample STBSUs had to meet the following conditions: (1) more than one year in operation, (2) at least one product or service, and (3) an income statement indicating profitability. These conditions tested viability, measured innovation, and demonstrated the validity of business model performance. The sample STBSUs represented different sectors and market segments to strengthen the probability of mainstreaming and validity of the results (Eisenhardt and Graebner, 2007) (Figure 4). Complete information regarding the sample is provided in Appendix 1.

### 3.2 Data collection

Data collection was carried out through face-to-face interviews for all French STBSUs and a combination of face-to-face and telephone interviews for Spanish STBSUs. The disruption caused by the outbreak of COVID-19 during the data collection between March and July 2020 led to the data being obtained via telephone interviews. All interviews lasted between 90 and 120 min. CEOs were the key respondents, as well as being the founders of the firms and often assumed the role of R&D managers as well. Following the data collection, the interviews were transcribed. Furthermore, two researchers were involved in this process to avoid interviewer bias, and the gathered information was validated by the respondents to ensure that the researchers’ understanding reflected the intended meaning of their responses.

Interview guidelines were used to standardise the conversations. The study was completed with two additional sources of information: a questionnaire with closed-ended questions based on the EU Innovation Scoreboard and OECD OI Scoreboard (Appendix 2), and secondary public information such as websites, news, social media, and/or internal documents provided by the STBSUs. According to Yin (1994), the combination of multiple sources confirms the validity and reliability of the research data. Additionally, this complementary information reduces the longitudinal approach to primary data collection (Hasche et al., 2017).

### 3.3 Data analysis

An important methodological decision regarding data analysis should be underlined. The replication approach was followed to determine behaviour and causal relationships. Therefore, each STBSU was analysed individually and in relation to each of the cases, cross referencing all of them, one by one, with the others. In addition, it allowed the identification of patterns in the cases analysed on the basis of the similarities and differences amongst all targeted STBSUs cross-checked in aleatory pairs until each STBSU had been cross-checked with the rest of the firms, following the methodological instructions in Eisenhardt (1989).

This iterative and progressive permutation supports the validity and reliability of this research (Yin, 2014) and ensures the thoroughness of the study, as highlighted in a similar research study by Hasche et al. (2017). This sequential and iterative process allowed the research team to understand the key role of stakeholders in implementing possible OI approaches and, consequently, better managing EO as a continuous challenge for STBSUs. The cross-check included a list of the main stakeholders, the manner of involvement, and the result of that collaboration in relation to OI and EO. The interviews were processed using Atlas.ti software to better systematise the analysis and enrich the results.

## 4 Results

An in-depth analysis of the empirical evidence found from the case studies was performed in order to obtain a broader perspective regarding OI and EO. The propositions are described and discussed consecutively as the connecting thread of the research questions. Qualitative research supports the results, but it should be emphasised that the constructs were introduced only if they showed common patterns in most cases. Therefore, the level of conformity of the relationships was validated through their coincidences. Consequently, the results can be generalised despite the need for further future research.

**Figure 1:**
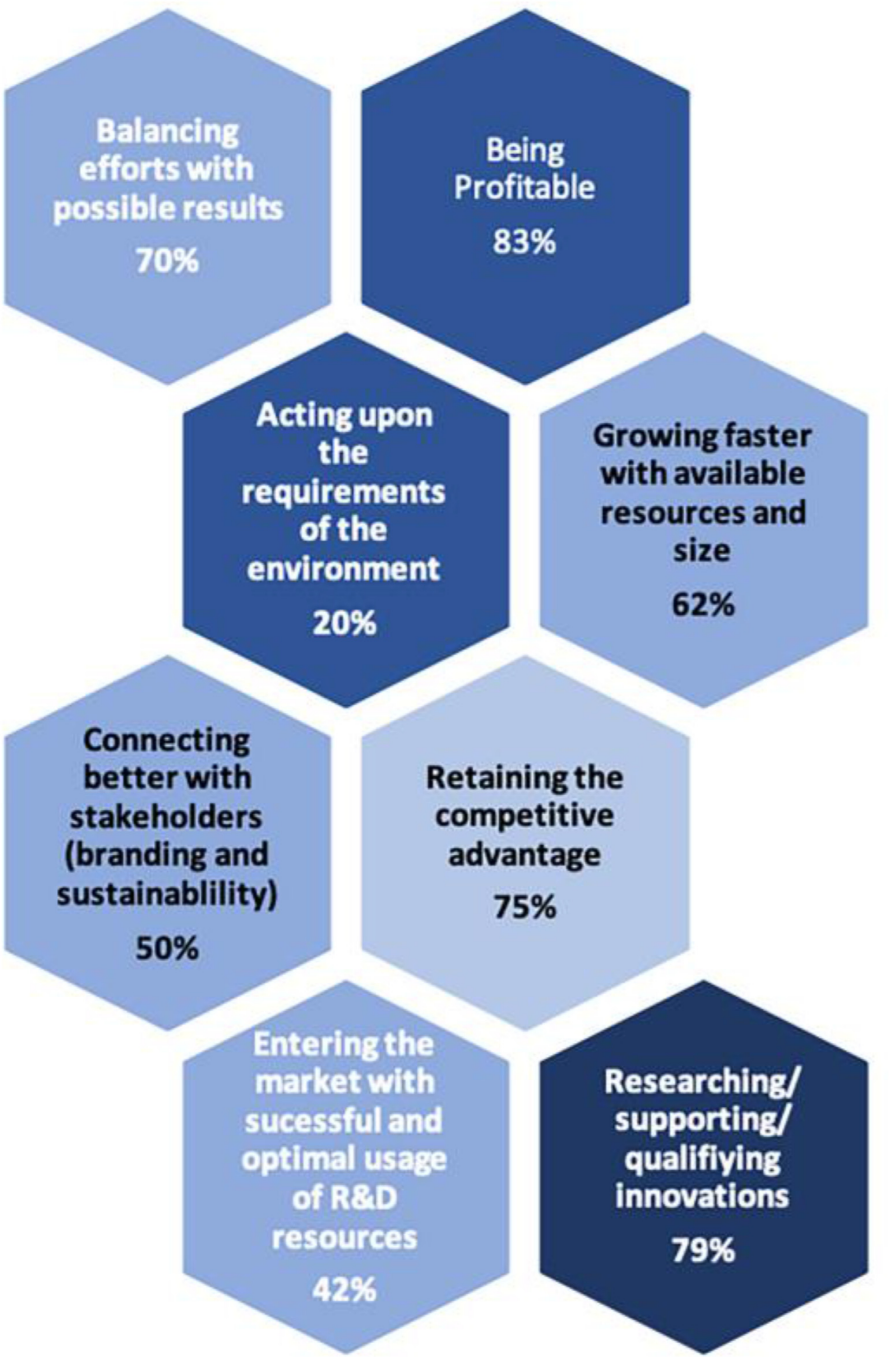
Reasons for stakeholder involvement in EO process.

The study was based on the belief that continuous management of EO is imperative for STBSUs’ survival and for competing with desirable levels of innovation. Thus, the question is how the OI approach helps these firms face this challenge through stakeholder involvement. All targeted STBSUs agreed on the importance of seizing and managing EO. Moreover, it transpired that the majority had a mechanism specifically for this task. Additionally, the same reasoning as discussed in previous sections was stressed in the interviews: (1) being profitable, (2) retaining a competitive advantage based on disruptive/radical innovations provided by the market, and (3) growth and survival (Figure 1). Furthermore, there was a wide consensus on the use of networks to achieve this. In particular, CEOs stated that collaboration with stakeholders is an effective way to balance internal effort in R&D projects while simultaneously enabling them to reduce the risks regarding the cutting-edge technology related to their business.

The following quotation represents the main motivation:

STBSU S “A company may not be competitive and not be profitable. It is essential to collaborate and involve the customers or else the company will fail”.

Respondents identified alertness to make up for their shortcomings. The following were the most referenced issues: firm size, young age of a company, consumers' lack of experience and knowledge of a company's innovations, and economic constraints regarding R&D activity. Accordingly, the following quotations illustrate the above-mentioned points.

STBSU A “I think STBSUs have common problems and a common way to solve them. An open mind to external people contributes to our goal of viability. We are new ventures, so our resources are limited at the beginning. R&D is not free and fund access is not easy; sometimes, our technology is not understood either by investors or by customers”.

STBSU E “We trust stakeholders and integrate them in our search for opportunities because our main obstacle is to achieve an income such that we fully benefit from our investments. Curiously, start-ups do not have problems with technology, the founders are really qualified, and we have a valuable background in the field, but marketing and financial skills are unknown areas. Beyond our technological product, we need to engage with customers and monetise our business model”.

STBSU T “It is curious, you do not have experience in the market, have little money and few people in your team but you have a clear and presumably brilliant idea so… you need other mechanisms to make opportunities a reality with guarantees”.

STBSU W “Is it not said that starvation sharpens inventiveness? We think that, in the case of STBSUs, our predisposition to listen to everybody is a consequence of our particular nature, is it not?”

It should be emphasised that STBSUs are regarded as innovative firms with a common characterisation. In other words, they have similar features and problems; however, some of the participants directly stated that EO is not exclusively for them. By contrast, opportunities are key for any new venture, but effective management becomes increasingly important for STBSUs because innovation is their source of differentiation and competitiveness.

Accordingly, the first proposition is:

### 4.1 Proposition 1

EO management is intrinsically linked with alertness and openness to the environment due to STBSUs' characterisation.

Uncertainty seems to be a central issue for STBSUs’ daily activities. Consequently, it is an undiscussed driver for OI implementation and a greater predisposition to involve stakeholders in the innovation process, recognising them as valuable sources of ideas, beta-testers, and even influencers on commercialisation strategies. The following statements illustrate this:

STBSU A “I wish I had a crystal ball; I would need it all the time. I lack some information to handle uncertainty, and the stakeholders help us with this stressful problem”.

STBSU N “When my environment told me I was completely crazy with my idea, I knew that the problem was to eliminate the uncertainty and the only way to do it was to ask my potential prescribers and users. Without their immediate enthusiasm, I would never be the entrepreneur that I am today.”

STBSU B “We have found a new opportunity on more than one occasion when testing or analysing the new requirements of our services with customers”.

STBSU H “We were clear about the field of opportunity, but the users of our solution really lead us to the current solution”.

STBSU J “I have specific examples of changes of direction in my business as a consequence of my external openness. In fact, at the beginning, I began with the idea that is radically different today. I realised that I needed to change my focus after discussing with outsiders, although undoubtedly the technology remains the same”.

STBSU S “It seems embarrassing, but our initial business model was completely different from the current one due to interaction with customers. The ‘disruptive’ technology did not change and it is our competitive advantage, but they led us to our present trading strategy”.

STBSU R “Well…our customers play a main role in improving our services and, in certain ways, some new products are the result of their involvement. The initial idea is ours, but it can be said that the current portfolio is shared”.

STBSU U “In my case, we develop a personalised solution for each customer, so I can say, each customer has an insight for the future developments of my firm”.

**Figure 2:**
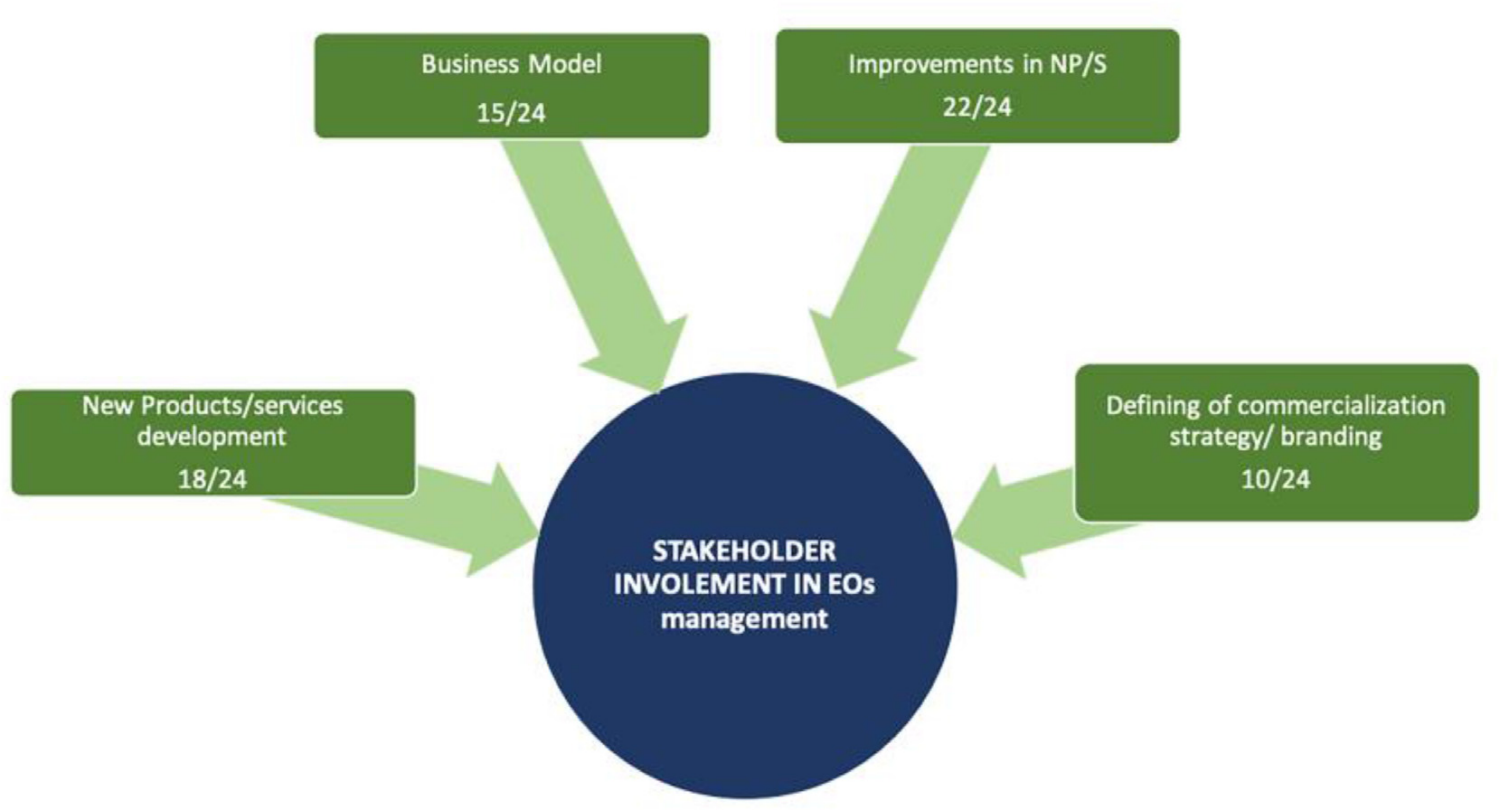
Impact of stakeholder involvement categorised by frequency.

Figure 2 shows the references made to various benefits of stakeholder involvement during the interviews.

The vast majority of CEOs underlined that innovation derived from collaboration can be incremental and disruptive. Stakeholders make it possible to identify new areas of application, functionalities, and different uses of products and services. Moreover, some STBSUs recognised that stakeholder involvement was the starting point for diversifying or setting new challenges of development.

Accordingly, the second proposition is:

### 4.2 Proposition 2

Stakeholder involvement resulting from the OI approach is a strategic mechanism for engendering new products, innovation projects, and viable business models of the EO.

**Figure 3:**
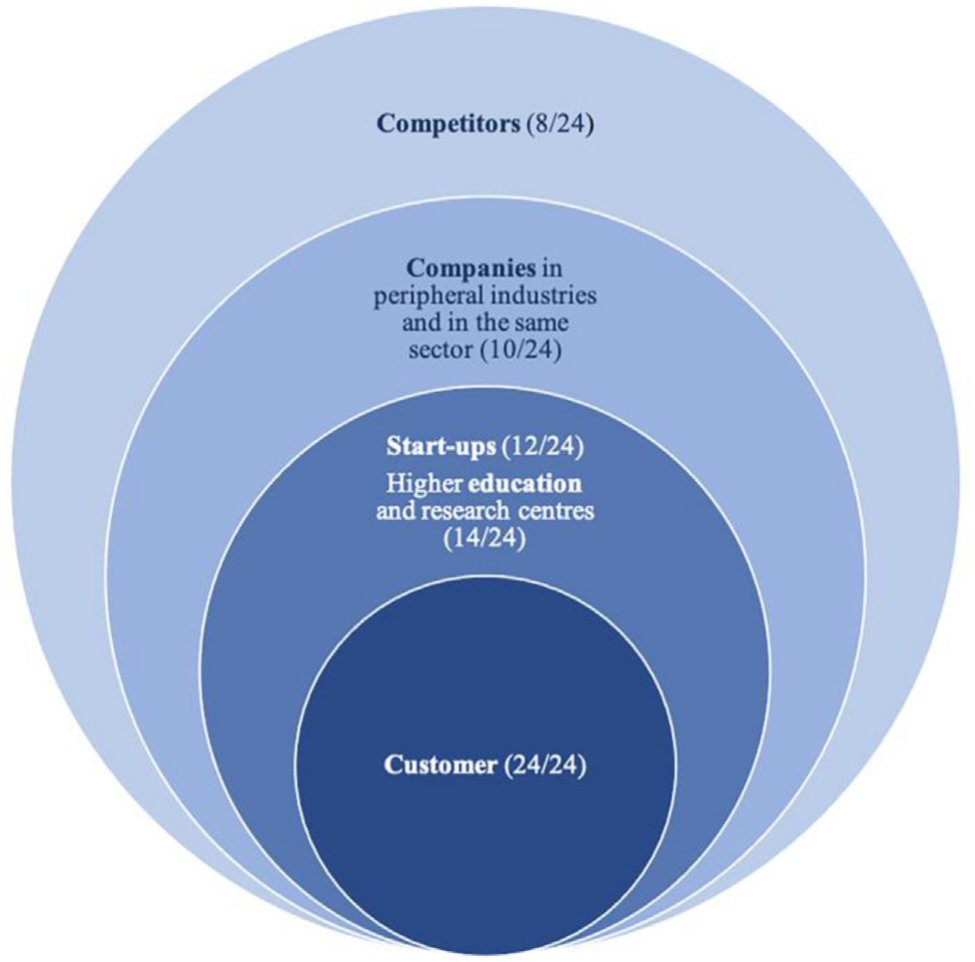
Types and numbers of stakeholders involved.

At this point, it is crucial to analyse: (1) who the most appreciated contributors are and (2) when and for what purpose stakeholders are involved. All the interviewees underscored customers as sources of new ideas, testers, and advisers regarding marketing strategies. However, differences between lead users and early adopters should be stressed. Both these players were particularly highlighted as valuable participants. Universities and research centres were frequently cited; these had a higher distribution among industrial and engineering and biotech STBSUs. The relationships with other STBSUs and the entrepreneurial ecosystems should be stressed, but their contributions were not so significant in the innovation process. They mainly helped in general management, highlighting the sharing of key contacts. Finally, it should be noted that Spanish and French STBSUs accounted for companies linked to their activity sector or even peripheral industries, but this was a more frequent practice for French firms. Influencers are also relevant, particularly for Internet-based start-ups. The least decisive participants were competitors, despite their continual benchmarking. Figure 3 shows the weightage of each stakeholder group.

**Figure 4:**
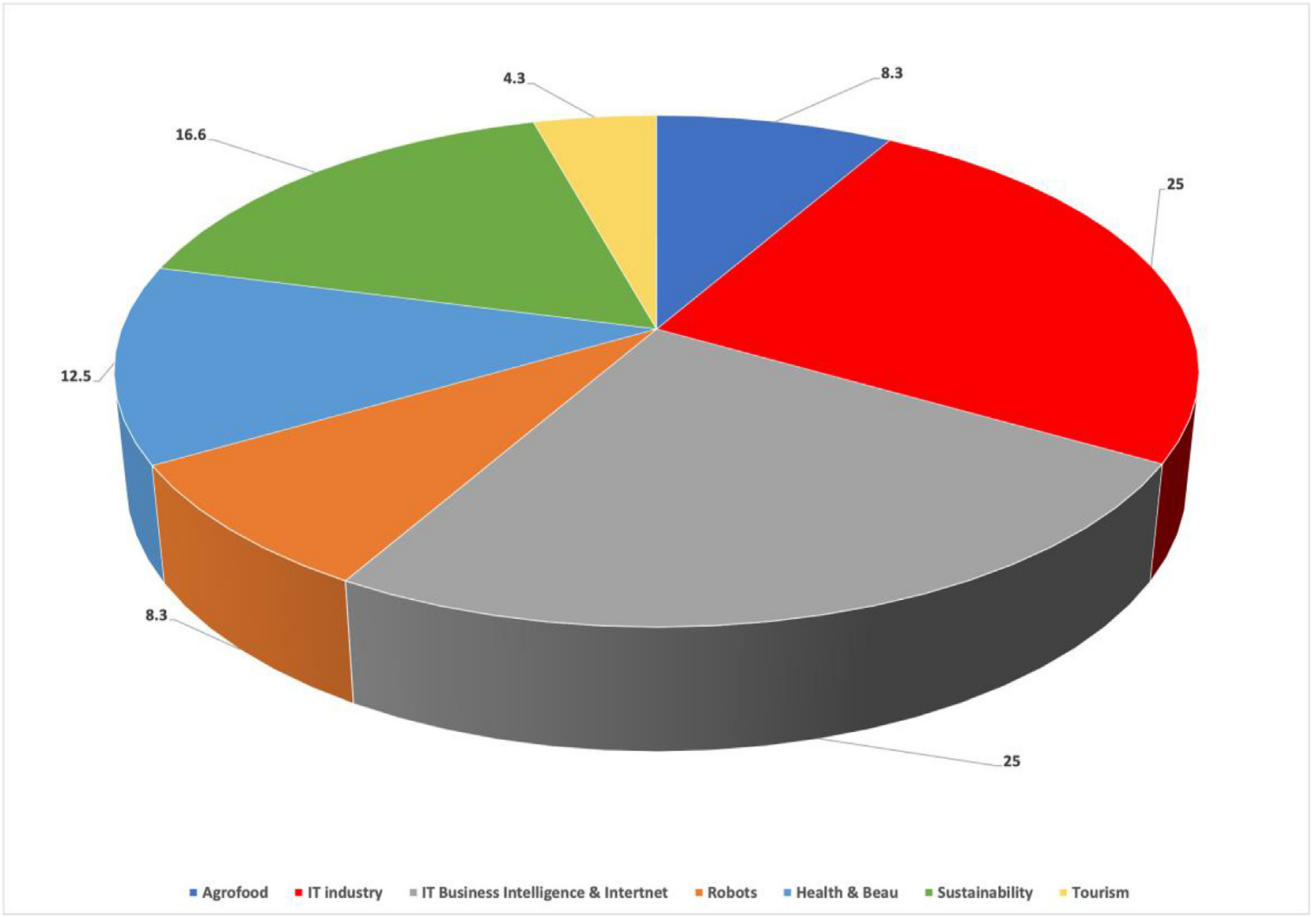
STBSUs disaggregated by activity sector.

Curiously, the evidence provides insight into the evolution of EO management with stakeholder involvement. Thus, networks are considered strategic, and they are progressively increasing.

Based on this evidence, the following proposition was introduced:

### 4.3 Proposition 3

Customer involvement provides the most added value for STBSUs from a broader perspective.

Regarding when and why stakeholders are integrated, a summary of quotations below best answers both issues:

STBSU C “Our dealers as the main customer, not the final customer, participate in the design and improvement of our products; they even help us evaluate how to introduce the product to market. We try to create a co-creation environment”.

STBSU G “They are involved in the design of the system not in the concept, especially in testing and improving it”.

STBSU L “The best proposals from the R&D department are analysed in a second phase in a workshop with customers. They give us their impressions and share their experiences about the products’ utility and potential and we act accordingly with regards to the development”.

STBSU M “We have an IT solution for the daily activity, so our customers were involved in the phase of design of the application and then as testers and a key group for defining and prioritising the improvement of our minimum viable product (MVP)”.

STBSU P “Our venture was risky, the sector was traditional and long established, our potential client base was big and apparently inaccessible, so we had to test the idea to continue or abandon the project. After the first model was validated, other questions emerged to define our strategy. For example, software as a service choice resulted from this interaction”.

STBSU V “We especially trust stakeholder involvement for commercialisation strategy and for the adaptation of the extra services related to our technology”.

This further explains how this link between EO and stakeholder involvement is carried out, and the interviews strongly indicate the OI approach as a driving force for stakeholder involvement. It was expressly referred to in 15/24 cases of STBSUs; in particular, 13 STBSUs had free and spontaneous OI as guides for their innovation management. Stakeholder involvement seems to be a logical consequence of this approach and a useful mechanism in the case of these firms.

## 5 Discussion

Despite extensive literature delving deeper into open innovation and innovation management in STBSUs, more empirical evidence is necessary to identify practices and strategies to achieve the competitiveness and survival of these companies. Therefore, this study analyses how entrepreneurial opportunities emerge as a result of stakeholder involvement and OI approach allows STBSUs to face the challenges derived from the particularities of their nature. As evidenced with the theoretical framework, there are not many studies focusing on the relations between EO with OI, so this paper, focusing on the role of stakeholder involvement, provides insights into how openness to external groups fosters the management of opportunities, which create new products/services and influence the business model. Specifically, an integrated framework based on an exploratory analysis of 24 STBSUs from France and Spain allowed us to better understand the key collaborators and driving forces in OI. In summary, stakeholder involvement seems to be an effective mechanism to implement this OI such that stakeholders become determinants of success for these kinds of firms in their challenge of overcoming uncertainty and maintaining a level of innovation owing to their continuous alertness regarding opportunities. The theoretical framework has already highlighted links and positive effect between innovation and social networks (Danarahmanto et al., 2020; Ollila and Yström, 2016) but the insights due the empirical evidence show stakeholders and how they are involved in innovation strategy to achieve the desired results in terms of innovation and feasibility. Moreover, exploration and exploitation of opportunities -concepts introduced by March (1991) and well-substantiated by Chesbrough (2003) for the OI approach-are more deeply analysed through specific practices and tools used by STBSUs targeted in the study. It should be noted that although the analysis was based on the whole sample of STBSUs and sub-samples composed of STBSUs from different countries, no differences were observed between the two.

This study's contribution to extant knowledge on entrepreneurship and OI is as follows. First, this study connects EO with OI and analyses stakeholders' involvement in a particular case of STBSUs, effectively bridging the gap in the theoretical framework, which existed due to a lack of evidence and contradictory results in previous literature (Hasche et al., 2017; Kirchberger et al., 2020; Michelino et al., 2017; Xie and Wang, 2020). In conclusion, OI literature emphasises and provides evidence on stakeholder contribution as an external source of innovation but, this study, also substantiates the focus on entrepreneurial opportunities contributing to a holistic understanding of how STBSUs evolve their strategy to pursue entrepreneurial opportunities. The patterns to implement stakeholder involvement, creating and capturing value for new product development, significant improvements of products and changes in business models, position this research work at the forefront with regard to theoretical framework. Furthermore, it searches for contact points and relationships between both fields (EO and OI). It is worth noting that in this regard the insights provided complement the contributions made by authors such as Jin and Shao (2022), Marcon and Ribeiro (2021), Eftekhari and Bogers (2015) and Xie and Wang (2020) and, specifically focused on the case of STBSUs.

Despite the fact that the findings of this study are in line with several previous research works that focused on the value of stakeholders such as Alberti and Pizzurno (2017), Chesbrough and Bogers (2014), Danarahmanto et al. (2020), Grimaldi et al. (2013), Jones & Banir (2019), Lassen and Laugen (2017), Ollila and Yström (2016), Randhawa et al. (2021), Shane et al. (2010), Secundo et al. (2020) and Urbinati et al. (2021), this study takes an important step, namely, it underscores the role of customers. However, extant studies such as Danarahmanto et al. (2020); Greco et al. (2016), Neyens et al. (2010), Oliveira et al. (2018) and Wouters (2010) have warned about the necessary balance between the required efforts to manage relationships with customers to achieve certain outcomes. In fact, the practices evidenced in this study show how STBSUs implement OI and involve stakeholders in New Product Development (NPD) as well as the main output of the collaboration being highlighted. In this respect, there are similarities in the way of developing co-creation environment for innovation management with the results of Ollila and Yström (2016). In fact, while there is extensive aforementioned literature focused on the importance of stakeholder management, this research work contributes by offering a holistic view on how and what performance are carried out.

However, the results do not deny that customers’ contributions have an impact on incremental or disruptive innovation (Ardito et al., 2020; Lassen and Laugen, 2017; Neyens et al., 2010; Randhawa et al., 2021), STBSUs stated that these are particularly significant for minimising risk due to their cutting-edge technology/innovation and for their sustainability, and thus agreed with authors such as Hasche et al. (2017); Hasan and Koning (2019); Marullo et al. (2018); Randhawa et al. (2021) and Urbinati et al. (2021). Furthermore, some similarities with Hannigan et al. (2018) and Lassen and Laugen (2017) were found. Thus, the level of disruption depends more on the management of the relationships with stakeholders and giving the role of co-creation to external sources so that customers can generate breakthrough products for firms.

Additionally, this study emphasises the contribution to EO management in fostering new or improved product development and shows further changes in business models, and determines commercialisation decisions as well. Specifically, previous literature has focused on these aspects individually, but does not pay attention to the positive effects and interactions between them for EO management. For example, Bogers et al. (2017), Chesbrough (2003), Danarahmanto et al. (2020), Eftekhari and Bogers (2015) and Sandmeier et al. (2010) stressed the positive influence on innovation, while de Oliveira & Cortimiglia (2017), Grama-Vigouroux et al. (2020), Mary George et al. (2016), Michelino et al. (2017), Shane et al. (2010) and Xu and Koivumäki (2019) underscored the support for the business model, and Keinz and Prügl (2010), Kirchberger et al. (2020) and Randhawa et al. (2021) highlighted the impact on marketability. In this sense, the results provide a more comprehensive view of the impact on performance and benefits of this approach. It should be emphasized that one of the main contributions of this study is the exploration of the connections between entrepreneurial intention and innovation in both directions.

The empirical evidence in this direction clarifies the knowledge in this field. Moreover, STBSUs can enhance the adoption of OI and the generation of stakeholder involvement in order to be more competitive and sustainable. Thus, the findings consolidate and widen the statements provided by recent research and according opportunities pointed out by West and Bogers (2017) in this field. The most highlighted issues are the uncertainty and value proposition to improve and lead the marketability of these new ventures. The uncertainty is in line with Ahn et al. (2017), Eftekhari and Bogers (2015), Eiteneyer et al. (2019), Greco et al. (2016), Kirchberger et al. (2020), Marullo et al. (2018) and Michelino et al. (2017). In fact, the predominant weight of uncertainty in the predisposition to manage EO with the support of stakeholder involvement is one of the main insights of the analysis by the research team in this study. Thus, agility, greater adaptability and safety in product development and in their introduction into the market are appreciated as key contributions in innovation management by STBSUs. Therefore, these insights allow researchers to take a step forward in the field of entrepreneurship regarding theories of entrepreneurial action, shedding light on the debate generated about creation and discovery of entrepreneurial opportunities (Alvarez and Barney, 2007; Foss and Klein, 2017; Jones and Barnir, 2019; Park, 2005) and connecting this background with how open innovation approach, involving stakeholders, can improve the opportunities to NPD, among others, in the case of STBSUS. As mentioned above, several authors have started this discussion thread (Alberti and Pizzurno, 2017; Grama-Vigouroux et al., 2020; Gruber et al., 2013; Secundo et al., 2020) but this study made an effort to show how these kind of companies put stakeholder involvement into practice.

## 6 Conclusions

This research sheds some light on the mediating role of stakeholder involvement in the relationship between OI and EO in STBSUs. Thus, it allows the literature combining both fields with empirical evidence of their connections to advance. The findings reveal that stakeholder involvement may cause the resulting opportunities to improve or give rise to new products, services, or changes in business models. Thus, RQ2 is answered specifically and it gives rise to a relevant contribution regarding the results prevailing in the literature which emphasize innovation output and performance but much less the value of co-creation taking into account stakeholders for innovativeness in STBSUs. Moreover, the analysis shows that STBSUs face EOs as a challenge related to their own nature, and they need mechanisms to manage them efficiently in order to compete and survive. Specifically, the practices evidenced, as well as STBSUs’ strong conviction about stakeholder contribution on their innovation, allow an answer to RQ1.

Accordingly, the OI approach, and in particular, stakeholder involvement, allow STBSUs to develop their activity successfully and sustainably. The predominant finding is in line with previous literature. However, this study also focuses on the association between motivations, stakeholders, and results, and thus makes a unique contribution to the field. First, STBSUs need a particular and specific focus, and further evidence is required. Second, the results provide interesting insights highlighting the role of customers regarding NPD and business models.

However, the vast majority of targeted STBSUs agreed that stakeholder contribution occurs both in incremental innovation and disruptive innovation. Additionally, the effect of stakeholder involvement on alleviating the pressure of uncertainty is related to the high level of innovation and lack of experience in the market. Thus, the influence of stakeholder involvement on the strategy of commercialisation is also shown.

### 6.1 Practical implications

The evidence provides a practical understanding of decisions, key agents, and connections between different mechanisms and consequences. Our findings enable CEOs to design strategies to maximise the outcomes derived from collaborative innovation with stakeholders by understanding the different contributions of each group. The STBSUs cases highlight broad agreement regarding which tools make stakeholder involvement easier: virtual community, panels of key groups, dynamics as a focus group or challenge for prescribers and lead-users are some of these. In fact, the convergence between digital and physical spaces of co-creation should be an issue to take into account for managerial decisions. Hence, the mechanisms and co-creation environment could work together to balance the effort and expected results.

### 6.2 Limitations and future research

The main limitations of this study are the specific focus on two entrepreneurial ecosystems, limited number of cases, and only one type of participant (CEOs) for gathering information in the interviews. Likewise, the methodological guidance shows the common pattern of behaviour or strategies of innovation management in STBSUs, but it does not allow identification if there are some sectors of activity which are more receptive to stakeholder involvement, even if there are some mediating or moderating effects for the adoption of this approach. Nevertheless, their joint analysis is methodologically justified because STBSUs are, due to their particular characteristics, a group of interest for the study, especially with regard to their level of innovation and their development and growth strategies (Gruber et al., 2013; Spender et al., 2017).

Despite these limitations, our results produced some valuable insights regarding how STBSUs face their own innovation challenges by staying alert with respect to EO and transforming it into beneficial outcomes, either for new products or business model dynamism. Therefore, we suggest that future research considers international comparisons, sector disaggregation and empirical tests contrasting models with large samples to extend the validity of the findings of this study and further generalise the insights it presents.

## Declarations

### Author contribution statement

Patricia P. Iglesias-Sánchez, Prof. Dr; Alain Fayolle, Prof. Dr; Carmen Jambrino-Maldonado, Prof. Dr; Carlos de las Heras-Pedrosa, Prof. Dr: Conceived and designed the experiments; Performed the experiments; Analyzed and interpreted the data; Contributed reagents, materials, analysis tools or data; Wrote the paper.

### Funding statement

Carlos de las Heras-Pedrosa was supported by Consejería de Economía, Innovación, Ciencia y Empleo, Junta de Andalucía [UMA18-FEDERJA-148].

Dr. Carmen Jambrino-Maldonado was supported by Universidad de Málaga. Spain [Funding for Open Access Charge:Universidad de Málaga/CBUA].

### Data availability statement

Data included in article/supp. material/referenced in article.

### Declaration of interest’s statement

The authors declare no conflict of interest.

### Additional information

No additional information is available for this paper.

## Figures and Tables

**Table:** 

STARTUP	INTERVIEWEE PROFILE	SECTOR	YEAR OF FOUNDATION	EMPLOYEES
A^1^	Founder and CEO	IT (agroindustry)	2016	3
	Higher education			
	Extensive experience in customer activity sector			
	No previous entrepreneurial experience			
B	Founder and CEO	Biotech (agroindustry)	2017	3
	Higher education			
	No previous entrepreneurial experience			
	Business idea is the result of research developed by the University promotional team (Doctorate Programme)			
C	Founder and R&D Manager	IT (industry)	2016	7
	Higher education			
	Extensive experience in the sector, wide range of networks with strategic partners for R&D and commercialisation.			
	High degree of specialisation in the key technologies of the product			
D	Founder and CEO	IT (industry)	2015	6
	Higher education			
	No previous entrepreneurial experience			
	Previous experience researching with similar technology in the doctorate programme			
E	Founder and MK Manager	IT (Internet)	2017	2
	Higher education			
	No previous entrepreneurial experience			
	No previous work experience in the same field although the founders have experience and knowledge as users			
F	Founder and CEO	IT (Business Intelligence)	2017	14
	Higher education			
	No previous entrepreneurial experience			
	Work experience related indirectly with the business project			
G	Founder and CEO	IT (industry)	2017	3
	Higher education			
	Extensive experience in the sector of activity			
	Wide range of networks with strategic groups and potential clients			
H	Founder and CEO	IT (e-health)	2016	7
	Higher education			
	No previous entrepreneurial experience			
	Extensive experience in the sector of activity and personal experience in the business concept			
	Wide range of networks with strategic users			
I	Founder and CEO	IT (industry)	2016	9
	Higher education			
	Previous entrepreneurial experience and extensive professional experience in high-tech and internet companies			
J	Founder and CEO	Robotic	2015	3
	Higher education			
	No previous entrepreneurial experience			
	Previous experience researching with similar technology in the doctorate programme			
K	Founder and CEO	IT (Business Intelligence)	2015	5
	Higher education			
	No previous entrepreneurial experience			
	High level of specialisation in key technologies due to previous experience			
L	Founder and CEO	Sustainable transport	2015	6
	Higher education			
	No previous entrepreneurial experience			
	Extensive experience in market entry with new products in big companies			
M	Founder and CEO	IT (Business Intelligence)	2017	14
	Higher education			
	No previous entrepreneurial experience			
	The whole promoter team stated to suffer the problem themselves.			
N	Founder and CEO	Health & Engineering	2016	2
	Higher education			
	No previous entrepreneurial experience			
	Direct contact with the problem that emerged the business idea			
O	Founder and CEO	Energy	2015	5
	Higher education			
	No previous entrepreneurial experience			
	Extensive experience in market entry with new products in big companies			
P	Founder and CEO	IT (Business Intelligence)	2017	3
	Higher education			
	No previous entrepreneurial experience			
	Work experience related indirectly with the business project			
Q	Founder and CEO	Sustainable construction	2017	3
	Higher education			
	Previous entrepreneurial experience			
	High level of specialisation due to previous research experience			
R	Founder and CEO	IT (Business Intelligence)	2018	2
	Higher education			
	No previous entrepreneurial experience			
	Previous experience in the sector and specialization in the key technologies and processes related with the service			
S	Founder and CEO	IT (Tourism)	2017	9
	Previous entrepreneurial experience and extensive professional experience in other fields			
T	Founder and CEO	Robotic	2016	21
	Higher education			
	No previous entrepreneurial experience			
	High level of specialisation due to previous research experience as Ph.D			
U	Founder and CEO	IT (industry)	2017	2
	Higher education			
	Previous entrepreneurial experience			
	High level of specialisation due their role as CEO in other STBSU			
V	Founder and CEO	IT (Industry)	2017	5
	Higher education			
	No previous entrepreneurial experience			
	Specialization derived from their final degree project and their master			
W	Founder and CEO	Cosmetics	2016	3
	Higher education			
	No previous entrepreneurial experience			
	Key specialization due to personal experience abroad			
X	Founder and CEO	IT (sustainability)	2018	4
	Higher education			
	No previous entrepreneurial experience			
	Business idea is the result of research developed at the University during their Doctorate Programme			

^1^ The anonymity of the participants was ensured changing the true names of the companies by a letter code.

**Table:** 

STBSU	SIZE		INNOVATION ACTIVITIES						IMPACTS	
			Innovators			Intellectual assets			Economic effects	
	Annual revenue (in €)	Employees	Percentage of expenditures in process innovations	Percentage of expenditures marketing or organisational innovations	Percentage of expenditures in new product development	PCT patent applications	Trademark applications	Design applications	Sales of product exports/international customer if it is a service	Sales of new products over total turnover
A	140,000	3	0%	0%	80%	0	0	0	Not export but 90% national 10% international customers	70%
B	20,000	3	25%	25%	30%	3 in progress (EU patent)	1 in progress	0	0	100%
C	560,000	7	5%	25%	75%	3 worldwide patent filed +2 in progress	1 in four classes (Eur/China/US/SriLanka	0	100%	100%
D	130,000	6	15%	5%	80%	0 0 (Industrial Secret and confidenciality contract)	3	0	Not export but 80% French customer although the most of them are multinational 20% international customer	100%
E	15,000	2	0%	5%	70%	0 (Software, especial limitations and regulations as PCT)	0	0	80% French and 20% from all over the globe (6.000 users in their community)	100%
F	200,000	14	5%	25%	10% the product is finished, at the beginning all the budget for new product development but in this stage, we are implementing and testing it.	0 (Software, especial limitations and regulations as PCT)	2	0	100% French customer although the most of them are multinational so their activity carry out all over the world, mainly in Europe.	100%
G	50,000	3	0%	0%	60%	1 in progress (worldwide patent)	1 in progress	0	Currently, all our clients are French. In fact, automotive industry is well represented by French companies like Citroen, Renault… but it is a strategic decision in the first stage. When we have the final system and it has been enough tested we will work in whole Europe and immediately after in USA and the rest of the world.	100%
H	150,000	7	0%	15%	85%	0	1	1	National customers	100%
I	50,000	9	10%	20%	60%	1 (in progress EUPCT)	1	0	100% national internautes community but currently they are working in reproducing the model in other countries	100%
J	70,000	3	60%	20%	20%	1 (Currently french but in short-term EUPCT)	1	0	100% national customer, although currently they are open markets in other countries (not only UE, highlight countries like Angola)	100%
K	300,000	5	0%	30%	70% (30% Research and 40% Development)	0 (Software, especial limitations and regulations as PCT)	1 in progress	0	100% International customer. Some of them are French but they are multinational companies with activity all over the world.	100% but it is possible differentiate between the product (solution and the basic implementation and each extra module and services of support of the solution depending on customer requirements. The last one are not considered by the Startup as new product)
L	120,000	6	20%	40%	40%	1	1	0	National	0% currently the incomes comes from consultancy but product is a demo/prototype
M	100,000	14	60%	10%	0%	0	1	0	National	All incomes come from the main IT solution
N	15,000	2	20%	15%	55%	0	1	0	National	100%
O	1,800.000	5	20%	20%	40%	0	1	0	National	100%
P	50,000	3	5%	5%	90%	1 (in progress)	0	1	60% National/40% international	100%
Q	125,000	3	20%	10%	70%	1	1	0	80% national 2% international	100%
R	13,000	2	20%	0%	80%	0	0	0	100%	100% MPV in the market just in forth month of 2019
S	165,000	9	30%	60%	10%	0	1	0	100%	100%
T	817,000	21	70%	25%	5%	0	1 EU	0	50% National 50% International	100%
U	215,000	2	15%	5%	80%	0	2	0	100% National	60% 40% consultancy
V	86,000	5	60%	10%	30%	0	1	0	100% National	70% 30% consultancy
W	950,000	3	40%	60%	0%	0	1	0	5% International 95% National	60%
X	34.,000	4	60%	10%	30%	0	0	0	100% National	100%
